# Glutamine-Glutamate Cycle Flux Is Similar in Cultured Astrocytes and Brain and Both Glutamate Production and Oxidation Are Mainly Catalyzed by Aspartate Aminotransferase

**DOI:** 10.3390/biology6010017

**Published:** 2017-02-24

**Authors:** Leif Hertz, Douglas L Rothman

**Affiliations:** 1 Laboratory of Brain Metabolic Diseases, Institute of Metabolic Disease Research and Drug Development, China Medical University, Liaoning 110000, China; lhertz538@gmail.com; 2 Magnetic Resonance Research Center, Radiology and Biomedical Engineering, Yale University, New Haven, CT 06520, USA

**Keywords:** aspartate aminotransferase, astrocyte culture, brain metabolism, glutamate-glutamine cycle, glutamate dehydrogenase, glutamate oxidation, glutamine synthetase, malate-aspartate shuttle, metabolic compartmentation, nitrogen balance

## Abstract

The glutamine-glutamate cycle provides neurons with astrocyte-generated glutamate/γ-aminobutyric acid (GABA) and oxidizes glutamate in astrocytes, and it returns released transmitter glutamate/GABA to neurons after astrocytic uptake. This review deals primarily with the glutamate/GABA generation/oxidation, although it also shows similarity between metabolic rates in cultured astrocytes and intact brain. A key point is identification of the enzyme(s) converting astrocytic α-ketoglutarate to glutamate and vice versa. Most experiments in cultured astrocytes, including those by one of us, suggest that glutamate formation is catalyzed by aspartate aminotransferase (AAT) and its degradation by glutamate dehydrogenase (GDH). Strongly supported by results shown in Table 1 we now propose that both reactions are primarily catalyzed by AAT. This is possible because the formation occurs in the cytosol and the degradation in mitochondria and they are temporally separate. High glutamate/glutamine concentrations abolish the need for glutamate production from α-ketoglutarate and due to metabolic coupling between glutamate synthesis and oxidation these high concentrations render AAT-mediated glutamate oxidation impossible. This necessitates the use of GDH under these conditions, shown by insensitivity of the oxidation to the transamination inhibitor aminooxyacetic acid (AOAA). Experiments using lower glutamate/glutamine concentration show inhibition of glutamate oxidation by AOAA, consistent with the coupled transamination reactions described here.

## 1. Introduction

In the adult brain neurons require metabolic collaboration with astrocytes in order to synthesize glutamate, the major excitatory transmitter, and its decarboxylation product γ-aminobutyric acid (GABA), the major inhibitory transmitter. The transport of glutamate and its precursor glutamine from blood to the brain is very slow and changes in plasma concentration of glutamine have only little influence on brain glutamate content [1,2]. The neurotransmitters glutamate and GABA must therefore be synthesized within the brain. Neuronally-released transmitter glutamate is also not re-accumulated into neurons to a significant extent, because one of the two main glutamate transporters, GLAST (EAAT-1) is completely, and the other, Glt-1 (EAAT-2) largely (80%–90%) localized on astrocytes [3]. Although the glutamate transporters EAAT 3–5 are neuronal, the total brain content of EAAT-3 is ~100 times lower than that of EAAT2 [3]. EAAT4 and EAAT5 transport glutamate slowly [3] and EAAT5 is present in the retina, but it has not been demonstrated in brain [3]. Part of the released GABA is also converted to glutamate [4] and accumulated by astrocytes [5,6].

**Figure 1:**
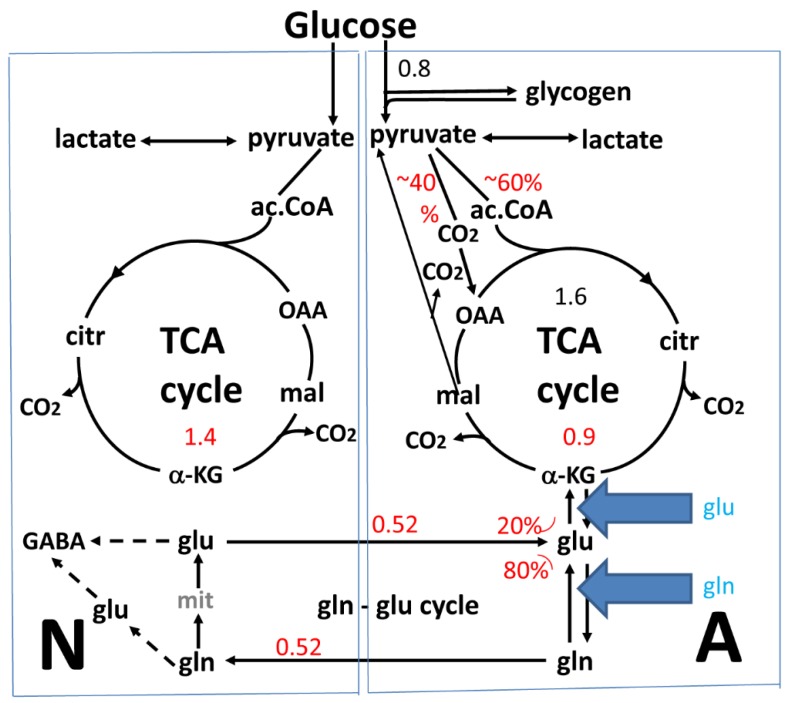
Cartoon of glucose metabolism via pyruvate in neurons (left—N) and astrocytes (right—A) and of glutamine-glutamate (γ-aminobutyric acid, GABA) cycling. This figure shows (i) metabolic pathways; (ii) metabolic rates (µmol/min per 100 mg protein or 1 g wet wt); and (iii) inhibition by excess extracellular glutamate or glutamine. One molecule of glucose is metabolized by glycolysis in the cytosol to two molecules of pyruvate in a complex and strictly regulated pathway (not shown). In both neurons and astrocytes pyruvate metabolism via acetyl coenzyme A (ac.CoA) leads to formation of citrate in the tricarboxylic acid (TCA) cycle by condensation with preexisting oxaloacetate (OAA), an end result of the previous turn of the cycle. Citrate oxidation in the TCA cycle includes two decarboxylations, resulting in re-formation of oxaloacetate, ready for another turn of the cycle, and reduction of NAD^+^ to NADH (and a single FAD to FADH_2_), leading to large amounts of energy (ATP) via re-oxidation in the electron transport chain. Pyruvate carboxylation, which is active in astrocytes, but not in neurons, creates a *new* molecule of oxaloacetate, which after condensation with acetyl coenzyme A, forms citrate that is metabolized in the TCA cycle to α-ketoglutarate (α-KG), which can leave the cycle to form glutamate (glu), catalyzed by aspartate aminotransferase (AAT). Further metabolism by the cytosolic and astrocyte-specific enzyme glutamine synthetase leads to the formation of glutamine (gln), which after transport to neurons is converted to transmitter glutamate or GABA in complex reactions (reviewed in [6]). Released transmitter glutamate is almost quantitatively re-accumulated in astrocytes, together with at least part of the released GABA (upper line of glu-gln cycle) and re-accumulated in the astrocytic cytosol. Here, 75%–80% is converted to glutamine and re-enters the glutamine-glutamate (GABA) cycle. The remaining 20%–25% is oxidatively degraded. This paper suggests that the default mechanism for the initial conversion of glutamate to α-KG is also transamination by AAT (see Figure 3), but it does not exclude a minor contribution by glutamate dehydrogenase (GDH). Operation of AAT in two opposite directions is thermodynamically possible since the reactions take place in two different compartments (cytosol and mitochondria) and also are temporally separate. α-KG is metabolized via malate, which can exit to the cytosol and be decarboxylated by cytosolic malic enzyme to pyruvate, which is oxidized in the TCA cycle via acetyl coenzyme A. Another possibility is that malate does not exit the TCA cycle but is further metabolized to α-KG after condensation with acetyl coenzyme A, allowing re-synthesis of another molecule of glutamate from only one molecule of pyruvate [6]. The degraded glutamate/GABA must in the long term be replaced by a quantitatively similar production of glutamate from glucose, in the first case by complete de novo synthesis from one molecule glucose, in the second from one half of a glucose molecule. However, temporary fluctuations in the content of glutamate occur. The initial part of GABA metabolism is different, as all GABA is metabolized via succinic semialdehyde, succinate and α-KG to glutamate. Numbers in black show rates re-calculated as µmol/min per 100 mg protein based on rate of glucose uptake in our own cultured astrocytes, and those in red are in vivo rates from [10]. The percentage distribution between metabolism via pyruvate carboxylation and acetyl coenzyme A is based on averages of results tabulated in [12]. Those for glutamate metabolism to either α-KG or glutamine are from [10]. The in vivo results that 40% of 0.8 µmol/min per 100 mg protein is metabolized via pyruvate carboxylation is consistent with the pyruvate carboxylation rates found by Kaufman and Driscoll [13] and overall the correlation between the metabolic rates in vivo and in culture is remarkably good, although other authors have described lower rates of glucose metabolism in cultured astrocytes. It should also be kept in mind that lightly anaesthetized mice have been used in most in vivo studies. Blue arrows: Suggested prevention of glutamate formation from α-KG and accompanying aspartate utilization by the presence of high extracellular concentrations of glutamate or glutamine, with the consequence that the AAT-mediated, coupled glutamate formation and degradation illustrated in Figure 3 can no longer operate. This appears to be the reason that so many studies have concluded that glutamate oxidation is catalyzed by GDH, whereas studies using low glutamate/glutamine concentrations find that AAT is involved. Modified from [6].

Neurons cannot synthesize glutamate, because it is produced from α-ketoglutarate (α-KG), which is a constituent of the mitochondrial tricarboxylic (TCA) cycle, and neurons are not capable of synthesizing TCA cycle constituents as it is an anaplerotic process. This is because they lack an enzyme, pyruvate carboxylase (Figure 1), which by condensing pyruvate with CO_2_ forms oxaloacetate, another TCA cycle constituent [7,8]. Astrocytes have high pyruvate carboxylase activity [7] and can accordingly synthesize glutamate and transport it, together with accumulated neuronally-released glutamate and GABA, after conversion to glutamine to neurons in a very active pathway: the glutamine-glutamate cycle. Above a low baseline value (at no brain activity) the adult brain cycle flux equals the neuronal rate of glucose utilization [9,10,11], which in turn corresponds to ~75% of the total rate of glucose utilization in both rat and human brain (reviewed and tabulated in [12]).

Both glutamate synthesis [13,14] and degradation, which also almost exclusively occurs in astrocytes, have repeatedly been studied in cultured astrocytes, but no attempt has previously been made to compare these processes quantitatively with the glutamine-glutamate cycle in brain. This will be done in the present paper. Moreover, there is no consensus regarding the enzyme catalyzing glutamate conversion to α-ketoglutarate, an essential first step in its degradation. Most authors assume that this process is catalyzed by glutamate dehydrogenase (GDH), but we have previously suggested that glutamate synthesis and degradation are coupled and both catalyzed by aspartate aminotransferase (AAT) [6,15,16]. This paper provides additional support for this concept by clarifying how AAT is capable of catalyzing the process in both directions. It also explains why so many studies, including some by one of us, previously have identified GDH as the enzyme involved in glutamate oxidation, probably because of interference with coupling between glutamate production and degradation by either high concentrations of glutamine or glutamate or by added ammonia.

In cultured astrocytes, the rate of pyruvate carboxylation is 3.4 nmol/mg protein per min at 2 mM extracellular K^+^. This rate increases by 50% at 10 mM K+ and 100% at 25 mM K+ [13]. The newly formed oxaloacetate is metabolized in the TCA cycle to α-KG, which can leave the cycle to form glutamate without depleting its normal contents of TCA cycle intermediates. Two types of brain enzymes are able to catalyze this process. One is the aminotransferases which exchange amino groups between different pairs of aminoacids/ketoacids. For example, aspartate aminotransferase converts α-KG to glutamate with simultaneous conversion of aspartate to oxaloacetate. Similarly, alanine aminotransferase catalyzes this conversion with concurrent conversion of alanine to pyruvate [17]. In the brain, AAT has a very high activity in both mitochondria and the cytosol [18]. However, as shown in liver, mitochondrial metabolism of glutamate by AAT invariably leads to the formation of aspartate [19]. In the brain, this reaction will not be able to remove glutamate as an excitatory amino acid, unless the generated aspartate is also disposed of. In this paper we will discuss and elucidate a previously proposed mechanism that does exactly that, because it couples astrocytic oxidation of glutamate and aspartate formation with a second phase in which de novo glutamate synthesis utilizes the nitrogen transferred to aspartate initially [6,15,16]. Glial nitrogen balance is maintained by removal of glutamate through conversion to glutamine. Moreover, astrocytic AAT activity is so active in the brain that the components of the reaction are near equilibrium in both astrocytes and synaptosomes [18,20]. This means that the AAT reaction alone would not be able to catalyze a continued production of glutamate, but the removal of the created glutamate by its conversion to glutamine secures that such a maintained synthesis can occur.

Although, as we will present, there is considerable evidence for such a critical role of AAT in both glutamate synthesis and oxidation it is still generally assumed that glutamate oxidation in brain is catalyzed by a second enzyme capable of bidirectional interconversion between α-KG and glutamate: glutamate dehydrogenase, which oxidatively removes NH_4_^+^ from glutamate or reductively condenses α-KG with NH_4_^+^. Its activity in adult brain and in astrocytes is much lower than that of AAT [21,22], but in contrast to AAT it operates without the need for any additional metabolic conversion, making it independent of the availability of aspartate/oxaloacetate, alanine/pyruvate or other transamination partners. In higher apes and humans two forms of GDH exist, GDH2 and GDH1. The latter is the enzyme also expressed in other species and its activity is allosterically regulated and seems to be induced by the need of the cell for ATP [23,24,25], shown by the ability of GTP formed via the TCA cycle to inhibit human GDH1 under extreme conditions of energy stress This inhibition is absent in GDH2, which previously was thought mainly to be expressed in astrocytes, but also has been demonstrated in neurons [26], similar to GDH1 [27]. This makes it uncertain whether the severe symptoms of GDH absence [28] primarily are due to astrocytic or neuronal deficiencies.

The conversion of glutamate to glutamine is carried out by glutamine synthetase, which in the brain is astrocyte-specific [29,30], although claims to a wider distribution have been made (reviewed in [30]). However immunochemical determinations of astrocytic enzymes and transporters [31] are fraught with difficulties [30,32] and although the main problem is lack of ability to discover relevant enzymes or transporters, false positive findings have also been reported [30]. Subsequently, glutamine is transferred to neurons in the glutamine-glutamate cycle together with astrocytically-accumulated, previously-released transmitter glutamate (see below). Most glutamine is accumulated by glutamatergic neurons, but some (about 20% depending on the brain region [2,4,33]) is taken up by GABAergic neurons where it is converted by glutamate decarboxylase (GAD) to GABA as previously reviewed [5,6,16,34].

In this paper we address in detail the identity of the main enzymes used for the initial step of astrocytic glutamate oxidation and the final step of glial glutamate synthesis and why this question has been disputed. We propose a two-stage model in which glutamate oxidation and synthesis are spatially and probably also temporally separated. This separation allows AAT to be used in both the oxidation and synthesis direction, and together with utilization of aspartate formed during glutamate oxidation in glutamate production, it eliminates concerns regarding accumulation of cytosolic aspartate except perhaps at highly elevated extracellular glutamate concentrations. This conclusion is therefore not in disagreement with a recent review by Cooper and Jeitner that AAT does not control the direction of the flow it mediates [18]. A comprehensive review of astrocytic cell culture data is consistent with this picture when it is taken into account that high levels of extracellular glutamate and glutamine may disturb the coupling by abolishing or reducing glutamate formation from α-KG. The role of GDH would then be to provide extra oxidative capacity needed only during extreme high activity conditions, or as extra synthesis capacity when ammonia under pathological conditions is highly elevated [18]. However, while extremely important functions of GDH in brain metabolism are presently being uncovered [23,24,26,28,35,36], they are unlikely to be related to its occasional and minor potential involvement in glutamate oxidation in the glutamine-glutamate cycle.

## 2. The Glutamate-Glutamine Cycle in the Brain In Vivo

A cycle carrying glutamate and GABA between astrocytes and neurons was first suggested by van den Berg et al. [37] and Benjamin and Quastel [38]. It is now known that one purpose of this cycle is to carry the glutamate which has been newly synthesized in astrocytes via glutamine to neurons, but a second and quantitatively major function is to mediate rapid transport of previously released transmitter glutamate and GABA to astrocytes and their return to neurons as shown in Figure 1. The enzymes necessary for the operation of the cycle are poorly expressed in neonatal brain, mature slowly, and in the rat the cycle and glutamatergic transmission only become fully operational at the age of one month [16,39,40]. In the mouse brain (of special interest because it used for many of the cultures discussed) the total rate of glucose consumption amounts to 0.7 µmol/min/g wet wt [41] which is rather close to that in rat brain. Only a minor part (20%–25%) of the glutamate flux from astrocytes to neurons and back again (Figure 1) represents newly synthesized glutamate. With astrocytes accounting for about one quarter of oxygen uptake in brain cortex, and up to 80% of astrocytic pyruvate metabolism occurring as glutamate formation/oxidation [12] the rate of glutamate synthesis must be between 0.1 and 0.2 µmol min per g wet wt. This is consistent with the conclusion that 20%–25% of the glutamine transported at a rate of ~0.5 µmol/min per g (Figure 1) from astrocytes to neurons in the glutamate-glutamine cycle is newly synthesized. The remainder of the flux (75%–80% of 0.7 µmol/min/g wet wt or about 0.5 µmol/min/g wet wt. (Figure 1) is made up of glutamine formed from transmitter glutamate and GABA which after their release are taken up in astrocytes and returned to neurons. Together these two fluxes, calculated from rates of oxygen consumption, are only slightly higher than the total glutamine-glutamate cycle flux determined by ^13^C NMR spectroscopy (Figure 1) in the lightly anesthetized rat [10]. The return of released transmitter GABA to glutamine is complex [4,6] and will not be discussed in the present paper. The fluxes in the two directions are approximately equal (Figure 1) and a truncated TCA cycle with build-up and release of aspartate is in vivo only seen during hypoglycemia, when conversion of glutamate-derived α-KG to oxaloacetate and aspartate provides some metabolic energy [42].

Consistent with the equal fluxes in both directions, the glutamate content in adult healthy brain is normally constant [43,44]. Accordingly, 20%–25% of the glutamate carried back to astrocytes is oxidatively degraded (a cataplerotic process) in astrocytes, where it serves as an important metabolic substrate [18,45,46,47,48] and in the process balances the anaplerotic de novo synthesis. Initially glutamate is converted to α-KG (with the roles of AAT and GDH respectively discussed in detail below), and α-KG circles to malate in the TCA cycle (Figure 1). Malate can either by continued circling in the TCA cycle be used for synthesis of a new molecule of glutamate (Figure 1) or it can exit into the cytosol and catalyzed by cytosolic malic enzyme, an astrocytic enzyme [49,50], form pyruvate, which then enters the TCA cycle for oxidative degradation [6]. Substantial conversion of pyruvate to lactate and subsequent lactate release has also been suggested [44]. However, this concept is partly based on experiments in cultured astrocytes [51], which release abnormally large amounts of lactate to the huge incubation volume. This also applies to glutamate-derived lactate [52].

In some cases rates of glutamate anaplerosis and cataplerosis are not completely identical, which can lead to transient increases in brain glutamate, e.g., during learning [53,54]. epileptic seizures [55,56], and during visual stimulation [57]. On the other hand, glutamate content in the occipital lobe is decreased by successful antidepressant therapy [58].

## 3. Glutamate and Glutamine Formation and Glutamate Oxidation in Cultured Astrocytes

Obviously the whole glutamate-glutamine cycle cannot be involved in cultured astrocytes (due to the absence of neurons), but in contrast to the in vivo studies, fixed concentrations of metabolic substrates (at least initially) can be used, and it is easier to use inhibitors and gene knockouts to investigate involvement of specific enzymes. Since glutamate is formed from α-KG, a TCA cycle intermediate, the rate of oxidative metabolism of glucose (the major metabolic substrate in brain and normally the substrate for cultured astrocytes) in the cultures is rate-limiting for glutamate production. Similar to the in vivo situation the rate of glucose metabolism can therefore provide information about that of glutamate synthesis. Respiration has been measured polarographically in cerebral astrocytes grown in primary cultures by Hertz and Hertz [59] and by Olson and Holtzman [60]. The former authors found a rate of O_2_ uptake of 300 µmol per h per 100 mg protein, which corresponds to 5 µmol O_2_ and thus oxidation of 0.8 µmol glucose per min per 100 mg protein, which approximately equals 1 g wet wt. [61]. This value is comparable to the rate of glucose utilization in mouse brain [41] and only two times higher than a value of 2.2 µmol O_2_/min per mg wet wt. determined in cultured rat astrocytes dissected from slightly different cultures and measured by micromanometry [62]. Olson and Holtzman [60] observed a slightly lower value, i.e., 1.7 µmol O_2_/min per g wet wt in their rat astrocytes. The cultures routinely used by Hertz and coworkers from 1977 and onwards (e.g., [45,63,64,65,66]) as well as in studies by the Schousboe/Waagepetersen group (e.g., [5,35]), the Sonnewald group (e.g., [14,67,68]) and with a slight variation by the McKenna group (e.g., [46,52]) express characteristics of their in vivo counterparts remarkably well [69]. Since most of the values for rates of glutamate metabolism that will be presented below were obtained using these cultures, the rate of glucose metabolism of 0.8 µmol glucose/min per g wet wt. is used in this paper (Figure 1). This would allow a similar maximum synthesis rate for glutamate if all glucose metabolism served to produce glutamate, but since this is not the case the actual rate for glutamate synthesis must be somewhat lower. Assuming that glutamate production in cultured astrocytes as in astrocytes in the brain accounts for 80% of astrocytic pyruvate metabolism [12] this means a glutamate synthesis of 0.64 µmol glucose/min per g *astrocytic* wet wt. With astrocytes accounting for 25% of brain cortical volume and oxygen consumption [12] this corresponds to between 0.1 and 0.2 µmol min per g *brain* wet wt, which is similar to the in vivo rate (Figure 1). This similarity may partly be coincidental because the glutamate production rate probably depends upon glutamate concentrations in the cells and in the medium. Normally there is no measurable glutamate in the medium, but since it does contain glutamine there is likely to be a continuous but rather slow production of glutamate which immediately is accumulated by the cells by the very potent astrocytic glutamate transporters [3].

Conversion of α-KG to glutamate in astrocytes could be catalyzed either by GDH or by a transaminase, most likely AAT. The question of which enzyme is used for this conversion is complicated by the virtually identical fluxes from α-KG to glutamate and from glutamate to α-KG, which would not be possible in a uniform aqueous solution. However, the cytoplasm does not constitute a uniform solution but is highly compartmentalized [70,71], although it allows inter-compartmental trafficking of metabolites and enzymes as well as trafficking between cytoplasm and organelles, including mitochondria. Furthermore, as discussed below there is likely a temporal separation in vivo between glutamate oxidation and glutamate synthesis due to continuous changes in glutamate concentrations during and between brain activations. Both spatial and temporal separation will affect thermodynamic directionality as discussed above.

The equilibrium constant for AAT is close to unity [18,72], making the reaction easily reversible, whereas for GDH reductive amination to glutamate is thermodynamically favored [73]. Nevertheless, Westergaard et al. [14] studying metabolism of 500 µM [U-^13^C] glutamate in glutamine-free medium to specific isotopomers in primary cultures of cerebral cortical astrocytes under control conditions and in the presence of the transaminase inhibitor aminooxyacetic acid (AOAA), clearly demonstrated that conversion of α-KG to glutamate occurs by transamination. This conclusion was reached by showing that AOAA abolished the formation of glutamate isotopomers, an indication of inhibited glutamate metabolism. However, under the conditions used (a high glutamate concentration) it had no inhibitory effect on an increase in the amount of citrate released from astrocytes in the additional presence of glutamate, indicating that there was no inhibition of α-KG formation from glutamate, which accordingly must have been catalyzed by GDH. The conclusion that glutamate formation from α-KG is mediated by transamination is in agreement with a low affinity of GDH for ammonia [74] and the extremely limited incorporation of administered ^13^NH_4_^+^ into the amino group of glutamate and glutamine in the brain in vivo compared to that into the amide group of glutamine [75]. The conclusions by Westergaard et al. [14] have been further supported by Sonnewald et al. [67,68], who also found that lactate labeling from glutamate was only slightly reduced by AOAA, indicating that GDH-catalyzed *oxidation* of glutamate was the major mechanism involved in the generation of α-KG during glutamate metabolism. This conclusion is in agreement with previous studies by Yu et al. [63,64] and Hertz et al. [65] shown in Table 1, who were the first to claim that glutamate oxidation is catalyzed by GDH because AOAA had little or no effect on CO_2_ production from glutamate. However, this result is in disagreement with those by Lai et al. [76] and Farinelli and Nicklas [77] who found a large inhibition. This difference is probably caused by different medium concentrations of glutamate or glutamine, as will be discussed later.

**Figure 2:**
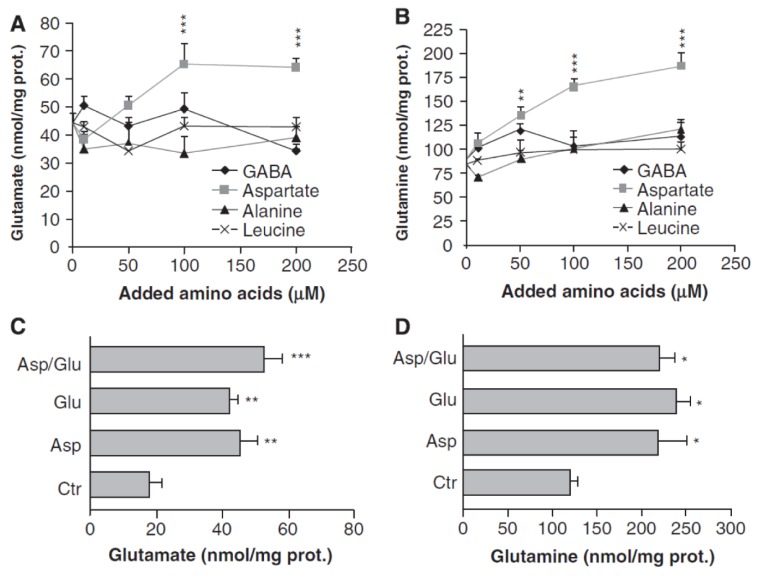
Aspartate promotes glutamate synthesis in cultured astrocytes, but only in the absence of extracellular glutamate. (**A**,**B**) Cortical mouse astrocyte cultures were incubated for 1 h in saline (in mM: 140 NaCl, 3.6 KCl, 0.5 NaH_2_PO_4_, 0.5 MgSO_4_, 1.5 CaCl_2_, 2 NaHCO_3_, 10 HEPES, pH 7.4) containing 2 mM glucose in the absence or presence of added amino acids (γ-aminobutyric acid, GABA, aspartate, alanine, or leucine; 10–200 μmol/L). Glutamate (**A**) and glutamine (**B**) contents were measured in cellular extracts and media by enzymatic methods. (**C**,**D**) Similar measurements in the absence of added amino acid (Ctr), the presence of 50 μM aspartate (Asp); 50 μM glutamate (Glu) or both together (Asp/Glu). Note that aspartate is the only amino acid among those studied which increases glutamate (**A**) and glutamine (**B**) and that the effect is abolished in the presence of glutamate (**C**/**D**). From [78].

An important study by Pardo et al. [78] investigated glutamate and glutamine formation in cultured astrocytes incubated with glucose as the metabolic substrate and treated with either aspartate, alanine, the branched chain amino acid (BCAA) leucine or GABA (Figure 2). Only aspartate, and not alanine, GABA, or leucine induced a large and significant increase in glutamine and glutamate synthesis. However, this effect disappeared when aspartate was added together with glutamate, an indication that 1) aspartate stimulates glutamate synthesis from α-KG, and 2) if extracellular glutamate is present, it dramatically decreases aspartate-mediated glutamate formation from α-KG. The possible route of synthesis suggested by Pardo et al. [78] was questioned by Hertz [15], who did however suggest an alternative route as discussed below. Nevertheless, the results by Westergaard et al. [14], Sonnewald et al. [67,68] and Pardo et al. [78] together provide strong evidence that glutamate synthesis from α-KG mainly occurs by transamination mediated by AAT, although a potential contribution from branched chain amino acids via their transaminase cannot be excluded [79]. Moreover, α-KG leads to a concentration-dependent increase in mitochondrial AAT activity, whereas addition of 5 mM glutamate significantly decreases AAT-mediated glutamate synthesis from α-KG in nonsynaptic mitochondria at 0.5 and 1.0 mM α-KG [80]. No rates of glutamate formation in the glutamine-glutamate cycle can be obtained from any of these studies, but addition of aspartate during 1 h was found by Pardo et al. [78] to increase the amount of glutamate by ~20 nmol/mg protein and that of glutamine by ~75 nmol/mg protein, suggesting an increase in glutamate formation from α-KG by at least 1.5 nmol/min per mg protein.

It is of interest to compare the rates found by Pardo et al. [78] to those that have been measured for glutamine synthesis from 50 µM glutamate, which in our cultured astrocytes amounts to 2.0–2.4 nmol/min per mg protein (Table 1) [63,81]. With the assumption of 10% protein in brain [61] this corresponds to 0.20–0.24 µmol/min per g wet wt. A higher protein content (200 mg/g wet wt) in our cultured astrocytes [82] would increase this value by a factor of two, but this may be irrelevant, since metabolism is more likely to be a function of cell protein than of cell size. It would, however, increase the glutamine formation rate to 0.40–0.5 µmol/min per g wet wt, a rate remarkably similar to the flux in the glutamate-glutamine cycle in vivo discussed above (Figure 1). Yudkoff et al. [83] found a four times higher rate of glutamine synthesis (Table 1), than those cited above, which they suggested could reflect the use of a higher glutamate concentration (250 µM). This suggestion is not consistent with the kinetics for glutamate uptake in astrocytes, i.e., a K_m_ of 10–20 or 50 µM [84,85] since the glutamate uptake should at most be two times faster at 250 than at 50 µM with this affinity. The young age (9 days) of the cells used in the Yudkoff study might further reduce glutamate uptake, which is slower in younger than in mature cultures [86]. It is therefore likely that the absence of glutamine for thermodynamic reasons may have caused the high rate of glutamine formation found by Yudkoff et al. [83].

On the other hand, Teixeira et al. [87] determined a somewhat lower rate of glutamine formation from [U-^13^C]glucose during long-term incubation of cultured astrocytes (~1 nmol/min per mg protein) by measuring increase in labeled glutamine at a time when there was no glutamate in the cultures. Earlier during the incubation, when glutamate was also present, the rate was twice as high. This might reflect direct synthesis of glutamine from glutamate, consistent with the glutamate-glutamine cycle in vivo carrying both newly-synthesized glutamate and previously released transmitter glutamate after its astrocytic accumulation. A caveat is that the cells used by these authors, like those used by Pellerin and Magistretti [88], were unable to derive energy by glutamate oxidation. With present knowledge about the importance of glutamate oxidation for brain function (see next sections) this represents an unexplained impairment of the cells.

Events in the glutamate-glutamine cycle leading to formation of transmitter glutamate after release of glutamine from astrocytes and its uptake in neurons are beyond the scope of this paper, but have previously been discussed in detail by the present authors [6,16]. Another subject which is mainly beyond the topic of this paper is the use of glutamine synthesis in brain astrocytes to ‘neutralize’ ammonia accumulated from the circulation. However, these rates are even during hepatic encephalopathy, when they amount to 15 nmol/mL [89], more than one order of magnitude below the flux in the glutamate-glutamine cycle of 500 nmol/g (Figure 1). There is also the big difference that ammonia detoxification under hyperammonemic conditions results in a net increase in glutamine [90,91,92,93], which is at least partly compensated for by glutamine release from brain [94]. In contrast, glutamine formation in astrocytes in the glutamate-glutamine cycle equals glutamine degradation in neurons, providing the possibility that all the ammonia needed for glutamate synthesis in brain might be supplied by ammonia released during conversion of glutamine to glutamate in neurons [6]. This concept is in agreement with the high influx of ammonia (> 3 μmol/min per 100 mg protein at 1 mM) into cultured astrocytes observed by Nagaraja and Brookes [95]. For these reasons we have in contrast to the review by Cooper and Jeinert [18] discussed the glutamine-glutamate (GABA) cycle independently of whole brain ammonia homeostasis. This does not mean that we disagree with the possibility that GDH-mediated glutamate oxidation might supply some of the NH_4_^+^, especially since small amounts of NH_4_^+^ may leave the brain due to efflux of glutamine [96] but rather that under most in vivo conditions GDH will play a minor role compared with AAT.

Table 1 also shows rates of CO_2_ production from glutamate. Most studies by Yu et al. and Hertz et al. [63,65,66] were carried out at a glutamate concentration of 50 µM (and normal medium glucose concentration [6–7.5 mM]) and maintaining the glutamine concentration of the medium (2 mM) in order to disturb ‘le milieu interne’ as little as possible. These studies all found CO_2_ production rates between 4.1 and 7.4 nmol/min per mg protein, measured as production of labeled CO_2_ from L-[1-^14^C]glutamate. A similar rate of oxidative metabolism of glutamate of (5.0 nmol/min per mg protein) was found polarographically by Hertz and Hertz [45] using 100 µM glutamate and no glutamine, and the metabolic rate was linear during a 60-min period. McKenna [46] found a slightly lower rate (1.4 nmol/min per mg protein with 1 mM glutamate), and before that Eriksson et al. [97] had found that addition of 100 µM glutamate to a medium containing glucose increased the respiratory rate with 5.0 nmol/min per mg protein.

**Table 1:** Glutamate metabolism in cultured astrocytes. AOAA: aminooxyacetic acid. Italicized value were obtained from cultures that had not been treated with dibutyryl cyclic AMP (dBcAMP). All other values are from cultures which had been treated during the culturing with dibutyryl cyclic AMP which has a differentiating effect on some but not all biochemical and physiological parameters [69]. * High concentrations of ammonia inhibit glutamate oxidation by ~50%. In neurons they also inhibit glutaminase [98] and thereby like elevated glutamine/glutamate must prevent coupling between glutamate production and degradation in the brain in vivo as discussed below.

Reference	Glu/Gln concentration	To glutamine (nmol/min per mg protein)	To oxaloacetate/aspartate (nmol/min per mg protein)	Glutamate to CO_2_ or polarographic assay (all nmol/min CO_2_ or O_2_ per mg protein)
Yu et al., 1982 [63]	50 µM/2 mM	2.4	1.1	4.1 ± 0.24 AOAA no effect
Yu and Hertz 1983 [66]	50 µM/2 mM			7.4 AOAA not tested
Yu et al. 1984 [64]	50 µM/0.5 mM			5.9 AOAA no additional effect when + ammonia at or above 1 mM *
Yudkoff et al., 1986 [83]	250µM/0 mM	9.0		
Hertz et al., 1988 [65]	50 µM/2 mM			5.1 AOAA not tested
Lai et al., 1989 [76]	190 µM/0 mM			2.7±0.22 AOAA: 0.74 ± 0.04; ammonia effect slightly less
Farinelli and Nicklas, 1992 [77]	50 µM/0.5 mM		*1.2*; 1.2 AOAA *0.1*; 0.1	6.0; 6.9 AOAA 0.3;0.6
Huang et al., 1994 [81]	50 µM/0.2 mM	2.0–2.2		
Hertz and Hertz, 2003 [45]	100 µM/0 mM no glucose			5.0 AOAA not tested
McKenna, 2012 [46]	1 mM/0 mM no glucose			1.4 AOAA not tested
McKenna et al., 1996 [52]	100–500 μM	Concentration-dependent; see text	Concentration-dependent, for details see text	

A previous study by McKenna et al. [52] compared metabolic fate of different glutamate concentrations using techniques similar to Westergaard et al. [14], and Sonnewald et al [67,68] but during a 2-hour incubation. The study showed that between 50 and 500 µM glutamate the formation of glutamine fell from constituting 85 to constituting slightly above 50 % of all metabolites, suggesting that in vivo return of incoming previously released transmitter glutamate via formation of glutamine is favored at lower glutamate concentration, whereas cataplerotic degradation occurs at higher concentrations. Total intracellular labeled glutamate increased 25 times between 50 and 500 mM when it reached 682 nmol/mg protein (~70 mM), but even at 50 mM less than one half was metabolized. TCA cycle metabolism of glutamate increased with increasing concentration and was almost equally divided between intracellular accumulation of labeled aspartate, metabolism of glutamate in the TCA cycle and extracellular accumulation of labeled lactate, which must have been formed from malate via pyruvate. Like the Sonnewald study [51], the McKenna study is a cell culture study explaining why lactate easily leaves the cells. Analogous to the large accumulation of non-metabolized glutamate found in this study [52], Huang et al. [81] found that after exposure to 50 μM glutamate intracellular glutamate rose to ~70 nmol/mg protein and only reached 10 nmol/mg protein (~1 mM) after 60 min. Thus, all glutamate concentrations used in Table 1 are high compared to the in vivo concentration. Moreover, a key difference in vivo from the cultured cells, which chronically were exposed to a high glutamate concentration (although it may decrease somewhat during the experiment due to the very intense glutamate uptake) is that astrocytes in vivo are far from a metabolic steady state. The glial endfeet surrounding synapses are repeatedly exposed to brief episodes of glutamate concentrations manifold higher than the average level in extracellular fluid of ~5 μM [99]. However between episodes of glutamate release extracellular glutamate drops dramatically and intracellular glutamate levels are decreased both due to continued oxidation and glutamine synthesis and subsequent export. Under this low glutamate condition, we propose that the net direction of glutamate metabolism is shifted to glutamate synthesis.

As described above, Westergaard et al. [14] and Sonnewald et al. [67,68], who used 500 µM glutamate showed that glutamate synthesis requires carbon supplied by the pyruvate carboxylase and nitrogen supplied by aspartate in an AAT-mediated reaction. However, they did not suggest any source for the aspartate needed for the AAT-mediated transamination and found that glutamate oxidation under the conditions used was catalyzed by GDH. Although the use of GDH during glutamate oxidation is supported by most, but not all, of the studies described below we suggest that this oxidation under most in vivo conditions is mainly catalyzed by AAT and that it provides the aspartate needed during glutamate synthesis. This is because the lack of inhibition of glutamate oxidation by AOAA shown in Table 1 only occurred in the presence of a pathophysiologically high concentration of glutamine or in the presence of high ammonia concentrations and because the experiments by Westergaard et al. [14] and Sonnewald et al. [67,68] used very high concentrations of glutamate. The suggested pathways for this coupled synthesis and oxidation of glutamate will be described in detail in the next section.

Yu et al. [63] who used a high glutamine concentration in the medium found no effect of AOAA on CO_2_ formation and therefore concluded that the reaction was catalyzed by GDH, a similar conclusion to that reached by Westergaard et al. [14] and Sonnewald et al. [67,68], who used a high concentration of glutamate. The high concentrations of either glutamate or glutamine used in all these studies are likely to have inhibited conversion of α-KG to glutamate or of glutamate to glutamine during glutamate transfer to neurons by its thermodynamic effects and potentially also by allosteric feedback. This must in turn inhibit AAT-mediated glutamate oxidation if the two processes are coupled, as will be discussed below. This explanation would be in complete agreement with a conclusion by Sonnewald et al. [68] that at low glutamate concentration glutamate is converted to α-KG by transamination, whereas the conversion at higher glutamate concentrations is catalyzed by GDH. It is also consistent with the finding by Pardo et al. [78] that the stimulation of glutamate and glutamine synthesis by aspartate is abolished when glutamate is present in the medium and with the inhibitory effect of a high glutamate concentration on AAT-mediated glutamate synthesis from α-KG in nonsynaptic mitochondria [80].

The concept of a glutamate/glutamine-inhibition of AAT-mediated glutamate oxidation is strongly supported by one study [76], which was performed in similar cells as those used by Yu Schousboe and Hertz and cited in Table 1, but in the absence of glutamine and at a somewhat higher glutamate concentration (190 µM—in order not to deplete the medium for glutamate during the experiment). This study showed a distinct and large inhibition of CO_2_ formation by AOAA. Even in the absence of AOAA the rate of CO_2_ formation was distinctly lower that those found at 50 µM glutamate (Table 1), but that could have been because the higher glutamate concentration, similar to what we have suggested for glutamine, inhibited glutamate formation from α-KG. As far as we know only one study by others [77] has reported absolute rates (Table 1). It used the same glutamate concentration as in our studies but only 0.5 mM glutamine and like Lai et al. [76] found that AOAA almost completed inhibited CO_2_ formation from glutamate. The studies by Lai et al. [76] and Farinelli and Nicklas [77] thus indicate that AAT is the major enzyme used for conversion of glutamate to α-KG during its oxidation under conditions relevant to the in vivo situation, where high glutamate concentrations only occur during brief time intervals. AAT-mediated transamination during glutamate oxidation is in complete agreement with studies on non-synaptic mitochondria [100] and on isolated whole brain mitochondria where glutamate is mainly metabolized (more than 75% of total metabolism) by transamination with oxaloacetate followed by oxidation of α -KG in the TCA cycle with GDH accounting for a major part of the remainder [17,21]. The findings by Lai et al. [76] and Farinelli and Nicklas [77] also support the conclusion that the high glutamine concentration used by Yu and coworkers [63,64] was a major reason why AOAA failed to inhibit glutamate metabolism. This is in agreement with the observation (Figure 2) that the stimulation of glutamate and glutamine production in cultured astrocytes by aspartate is abolished in the presence of extracellular glutamate [78]. Unfortunately AOAA inhibition in reference [64], when a low concentration of glutamine was used that should have allowed AAT mediated glutamate oxidation, was only studied in the presence of a high concentration of ammonia. High ammonia concentrations will lead to net glial glutamate formation [81] via anaplerosis and GDH (and in vivo they will also interrupt neuronal glutamate production [98]) thereby interfering with the normal coupling between glutamate oxidation and resynthesis needed to maintain the glutamate/glutamine cycle.

Prolonged exposure of astrocytes to extremely high glutamate concentrations would only occur in exceptional circumstances in vivo such as complete depolarization or intense firing during seizure, rendering the conclusions of the importance of GDH in many tissue culture studies mainly relevant to extreme physiological to pathological conditions. This also applies to the studies by Yu, Hertz, and Schousboe cited in Table 1 who used a high concentrations of glutamine, which is likely to have a similar effect as glutamate 

## 4. Suggested Pathway for Coupled Formation and Oxidation of Glutamate

**Figure 3:**
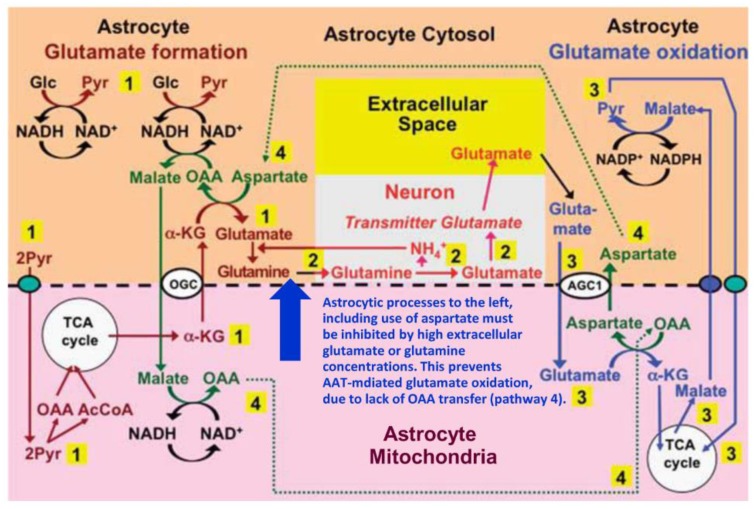
Proposed pathway for coupled production and metabolism of transmitter glutamate using aspartate transamination for exchange between α-KG and glutamate. Joint pyruvate carboxylase and pyruvate dehydrogenase activation generates a “new” molecule of citrate (lower left corner) as detailed in Figure 1. Citrate-derived α-KG exiting the mitochondrial membrane leaves the astrocytic TCA cycle and is transaminated with aspartate to form glutamate, with concomitant oxaloacetate (OAA) formation from aspartate. The mitochondrial exit of α-KG occurs via the α-ketoglutarate/malate exchanger, generally acknowledged to be expressed in astrocytes, and the cytosolic malate with which it is exchanged, is generated via NADH-supported reduction of oxaloacetate generated from aspartate. Glutamate is amidated to glutamine (pathway 1), which is transferred to glutamatergic neurons (without indication of any extracellular space in the Figure). High extracellular concentrations of glutamate or glutamine (blue arrow) will at least in cultured astrocytes make the astrocytic production of glutamate and glutamine unnecessary (and/or prevent the reaction for thermodynamic reasons) and thereby inhibit glutamate formation from α-KG and the associated transamination of aspartate. In neurons glutamine is in a complex pathway converted to glutamate, accumulated in vesicles and released as transmitter glutamate (pathway 2). Subsequent reuptake of glutamate and oxidative metabolism in astrocytes (pathway 3) is normally of similar magnitude as the production of glutamate described above. Cytosolic glutamate is transferred to mitochondria via the aspartate-glutamate exchanger AGC1 (aralar) in exchange with mitochondrial aspartate generated from OAA formed during the synthesis of glutamate (pathway 4) and re-converted to aspartate during transamination of glutamate to α-KG. In turn, the cytosolic aspartate is used during glutamate synthesis in the transamination of α-KG to glutamate in pathway 1 after transfer via pathway 4. However, when glutamate production from α-KG is inhibited by high extracellular concentrations of glutamate or glutamine (blue arrow) or by excess ammonia (inhibiting the neuronal glutaminase [98]), the exit of aspartate to the cytosol in exchange with glutamate during glutamate oxidation will no longer be compensated for by aspartate use during glutamate formation. This abolishes glutamate oxidation via AAT, so that formation of α-KG must be catalyzed by glutamate dehydrogenase (GDH). This is unlikely normally to take place in vivo since elevated extracellular glutamate only occurs during bursts of neuronal activity and glutamate uptake and glutamine synthesis rapidly clear extra- and intracellular glutamate. Metabolism of α-KG is only shown via malate exit and pyruvate formation. Since this is an oxidation in the cytosol it must be followed by malate aspartate shuttle (MAS)-mediated transfer of a reducing equivalent to the mitochondria. Biosynthesis of glutamine is shown in brown and metabolic degradation of glutamate in blue. Synthesis of glutamine and export from the glia is critical since it restores nitrogen balance (otherwise concentrations of glutamate and aspartate would continuously rise). Redox shuttling and astrocytic release of glutamine and uptake of glutamate are shown in black, and neuronal uptake of glutamine, hydrolysis to glutamate, and its release is shown in red. Reactions involving or resulting from transamination between aspartate and oxaloacetate (OAA) are shown in green. Small blue oval shows pyruvate carrier into mitochondria and small purple oval malate carrier out from mitochondria. AGC1, aspartate/glutamate exchanger, aralar; α-KG, α-ketoglutarate; Glc, glucose; Pyr, pyruvate; OGC, malate/α-ketoglutarate exchanger. AGC1 is for graphical reasons only indicated during the initial part of glutamate oxidation, where it constitutes part of the suggested pathway, but not where it is generally acknowledged to function in MAS, i.e., during synthesis of pyruvate from glucose (pathway 1) and from malate (pathway 3). The glutamine–(GABA) cycle is accordingly extremely dependent upon the abundant expression of AGC1 in astrocytes described above. However, if malate generated during glutamate degradation does not exit the TCA cycle but is further metabolized to α-KG, allowing re-synthesis of another molecule of glutamate from only one molecule of pyruvate and abrogating pyruvate formation from malate (Figure 1) trafficking via AGC1 will be considerably reduced, although certainly not abolished. Slightly modified from [15].

As shown in Figure 3, we have previously suggested a model according to which conversion of oxaloacetate to aspartate during glutamate oxidation (after its entry into mitochondria via the glutamate/aspartate exchanger AGC1) creates the aspartate needed for de novo glutamate formation from α-KG [6,15,16]. This model was triggered by the observation by Pardo et al. [78] that aspartate as the only amino acid studied increases glutamate formation from glucose in cultured astrocytes. Key points in the model are (1) that during glutamate synthesis the reduction of oxaloacetate to malate, which reverses the reduction of NAD^+^ to NADH during glycolysis (when glyceraldehyde-3-phosphate is converted to 1-3-biphosphoglycerate), is followed by malate entry into mitochondria and its conversion to oxaloacetate (OAA) when NADH is re-oxidized to NAD^+^; (2) that during glutamate degradation the same molecule of OAA is transaminated to aspartate when glutamate, after its entry into mitochondria in exchange with aspartate is transaminated to α-KG; (3) that the generated α-KG enters the TCA cycle, which drives the series of reactions forward, (4) whereas the aspartate molecule re-enters the cytosol, where it again supplies the aspartate needed for transamination of α-KG during the synthesis of glutamate. It is critical to note that AAT-mediated conversion of glutamate to α-KG occurs in mitochondria and that the generated aspartate travels from mitochondria to cytosol via the glutamate-aspartate exchanger/AGC1/aralar (coded by *Slc25a12*). In contrast, AAT-mediated glutamate synthesis occurs in the cytosol, with malate derived from aspartate via oxaloacetate travelling from cytosol to mitochondria via the malate/α-KG exchanger OGC (coded by *Slc25a11*). AGC1 (aralar) is also required for the malate-aspartate shuttle (MAS) activity needed to carry a reducing equivalent to the mitochondria following the cytosolic oxidation of malate to pyruvate (shown in the upper right corner of the Figure) and for the pyruvate production from glucose (shown at the far left of the Figure), but for graphical reasons this is not indicated. It is therefore important that aralar is abundantly and lastingly expressed both in astrocytes obtained directly from the mouse brain by fluorescence-activated cell sorting and in well-differentiated astrocyte cultures. At least as high mRNA expression in astrocytes as in neurons was first demonstrated in the freshly isolated cells by microarray analysis [101]. Subsequently this was confirmed in similar cells and in well-differentiated astrocyte cultures by the more accurate real-time polymerase chain reaction (PCR) and similar aralar protein expression was shown by western blotting using a monoclonal anti-aralar antibody [102].

The high expression of aralar protein and mRNA in astrocytes freshly obtained from the brain [101,102] is especially important for any mechanism depending on operation of the malate aspartate shuttle. The presence of aralar in astrocytes has been controversial, mainly due to inability by several authors to demonstrate aralar immunochemically [103,104]. However, as previously mentioned [32] this is the case for many astrocytic proteins. Also, using a very careful immunochemical analysis astrocytic aralar has been detected [78], and although it was in lower amounts than in neurons this finding greatly supports the high protein and mRNA expression for aralar found in freshly isolated astrocytes [101,102]. A recent review [105] re-opens this debate and further supports the claim that astrocytes are deficient in aralar by pointing towards the observation that exogenous aspartate is able to stimulate synthesis of glutamate and glutamine even in aralar−/− astrocytes [78]. However, aralar is not involved at the stage where added aspartate stimulates synthesis of glutamate and glutamine (Figure 3) and α-KG can be made from added aspartate. Therefore it makes no difference whether wild-type or aralar−/− astrocytes are used. Further evidence for plentiful astrocytic aralar expression are cell culture studies showing similar rates of oxidative glucose metabolism in neurons and astrocytes (Figure 1) and the large number of in vivo studies showing high glucose oxidation in rodent and human brain (e.g., [4,10,12,39,43]).

In addition to a fully functioning malate/aspartate shuttle, prevention of excessive accumulation of aspartate and glutamate requires as previously mentioned that glutamine synthesis is active in the cytosol. One amino group is introduced into aspartate during mitochondrial oxidation of glutamate and returned to glutamate during its formation in the cytosol, with the two processes coupled by the transport of aspartate. It is of critical importance that the generated glutamate is further metabolized to glutamine in order to maintain nitrogen homeostasis. Without glutamine synthesis to remove the NH_4_^+^ equivalents brought into the glia by glutamate uptake, the levels of asparate and glutamate will rise and eventually prevent the use of AAT for oxidation. The association between aspartate and glutamine synthesis is supported by the observation that exposure of cultured astrocytes to 3 mM ammonia decreases the aspartate content from 7.0 to 2.6 nmol/mg protein within 30 min [76]. This decrease can be explained by the increased glutamine synthesis, which causes a decrease in glutamate content that to some extent is counteracted by an increased glutamate synthesis [106,107]. It suggests that formation of glutamate, which was not coupled to glutamate oxidation, at least partly used the intracellular pool of aspartate to perform glutamate synthesis, which accordingly was AAT-mediated. This observation supports our proposal that AAT has a major role both in deamination of glutamate to α-KG as the first step in glutamate oxidation and in the amination of α-KG to glutamate in the subsequent replacement of oxidized glutamate by anaplerosis, and that the directionality of the processes is established respectively by TCA cycle activity and glutamine formation. The use of AAT in both directions could be seen as creating a thermodynamic paradox, since under steady state conditions there can only be one direction of net flow. However, this potential argument against AAT activity is invalid because of spatial separation between cytosolic glutamate formation and mitochondrial degradation, removal of synthesized glutamate by glutamine formation and of α-KG by TCA cycle activity as well as temporal separation during exposure to high and low extracellular glutamate concentrations shown by modeling. A recent study performing stoichiometric modeling concluded that the well-known increase in glycolysis compared to oxidative metabolism (decreased OGI) during brain activation can be partly (but not completely) explained by an increase in glutamate-glutamine cycling [108], with a doubling of cycle flux when the ratio between rates of oxidative and glycolytic metabolism decreases from 5.5 to 4.25. This indicates that cycle activity must vary greatly from moment to moment in concert with brain activation or lack thereof.

## 5. Evidence from GDH Knock Down/Inhibition Studies of the Roles of AAT and GDH in Glutamate Oxidation and Resynthesis

As reviewed above several studies, primarily of astrocyte cell cultures, have proposed an important role of GDH. However they have based their conclusions on the lack of effect of AOAA, which may be misleading in the presence of substantial amounts of extracellular glutamate or glutamine. More recently glial cell cultures with knockouts or knockdowns of GDH have been studied to more directly test the roles of GDH and AAT. Frigerio et al. [109] found a reduction of glutamate oxidation on the order of 30%, although this is was in the presence of 250 µM glutamate, which was interpreted as supporting a key role of GDH. However, the remaining glutamate oxidation supports a highly significant role of AAT, even in the presence of the relatively high glutamate concentration in the medium which might have caused a similar inhibition as in cultures with normal GDH function. Skytt et al. [110] used a lower glutamate concentration, but the presence of 2.5 mM glutamine in the medium may explain the abnormalities observed in astrocytes from GDH-deficient astrocytes. In brain slices (which have a reduced rate of metabolism compared to the active brain even when stimulated [111,112]) from mice with a GDH knockout, Frigerio [109] et al. found no impact on the glutamate/glutamine cycle (assessed electrophysiologically) but an in vivo alteration in glutamine levels consistent with a perturbation of GDH’s role in ammonia and glutamine metabolism. This, again, supports a primary role for AAT in the entire glutamine-glutamate cycle. Unfortunately, in vivo studies using ^13^C labeling which could address this issue more conclusively have not been published.

## 6. Conclusions

It is a paradox in brain metabolic studies that astrocytes simultaneously oxidize and produce glutamate. Several authors have previously found evidence that the dual role was accomplished by the use of two different enzyme systems, GDH for oxidation and AAT for re-synthesis, and one of us (Leif Hertz) is an author of the first study claiming GDH-mediated oxidation [63]. Most evidence for this mechanism is based on astrocyte culture studies in which AAT inhibition did not eliminate glutamate oxidation. However a detailed re-analysis of the cell culture literature shows that the lack of effect of AAT inhibition is primarily observed when the extracellular concentrations of glutamate and glutamine are un-physiologically high or when highly elevated ammonia concentrations are present, whereas the cellular metabolic rates of the cultures themselves are remarkably similar to those of astrocytes within the brain. Under more physiological medium conditions AAT inhibition can dramatically reduce glutamate oxidation. We have interpreted these observations, together with several previous studies in mitochondria, as suggesting transamination as the default mechanism for both glutamate synthesis and oxidation, but retaining a role for GDH under specific conditions, which may include high levels of extracellular glutamate, accumulation of ammonia or oxidation in astrocytes far apart from those in which it was synthesized. We believe that the suggested model should be able to function, and that the possibility that additional mechanisms such as those suggested in [18] are also required can only be tested by in vivo experiments. The increased availability of animal knockouts and knockdowns of relevant enzymes and ^13^C and ^15^N labeling methods should make such experiments possible, including establishing the use of AAT and GDH in the glutamine-glutamate (GABA) cycle under different conditions. A number of other, related characteristics of the cycle have previously been discussed elsewhere, e.g., the mechanisms triggering the activation of this cycle and its close association with neuronal rate of glucose utilization and the similar astrocytic-neuronal and neuronal-astrocytic fluxes. Both Patel et al. [113] and we ourselves [6] have debated reasons for the correlation between cycle flux and oxidative metabolism. The level of extracellular K^+^ was found to regulate pyruvate carboxylation in cultured astrocytes [13] and high extracellular K^+^ concentrations might activate glutamate synthesis, whereas the glutamate concentration is likely to regulate glutamate oxidation, at least within a certain range (determined by the K_m_ and V_max_ values for its uptake), as also concluded by Michael Robinson and coworkers [114]. Coupling between glutamate production and degradation will contribute to equalize fluxes from astrocytes to neurons and from neurons to astrocytes.
